# Athlete's Heart Assessed by Sit-Up Strength Exercises in Military Men and Women: The CHIEF Heart Study

**DOI:** 10.3389/fcvm.2021.737607

**Published:** 2022-01-26

**Authors:** Yu-Kai Lin, Kun-Zhe Tsai, Chih-Lu Han, Jiunn-Tay Lee, Gen-Min Lin

**Affiliations:** ^1^Department of Internal Medicine, Hualien Armed Forces General Hospital, Hualien City, Taiwan; ^2^Institute of Medical Sciences, National Defense Medical Center, Taipei, Taiwan; ^3^Department of Neurology, Tri-Service General Hospital, National Defense Medical Center, Taipei, Taiwan; ^4^Department of Dentistry, Tri-Service General Hospital, National Defense Medical Center, Taipei, Taiwan; ^5^Department of Medicine, Taipei Veterans General Hospital, Taipei, Taiwan; ^6^Department of Internal Medicine, Tri-Service General Hospital, National Defense Medical Center, Taipei, Taiwan

**Keywords:** athlete's heart, cardiac remodeling, muscular strength exercise, left ventricular diastolic function, sex differences

## Abstract

**Background:**

Greater changes in cardiac structure and function in response to physical training have been observed more often in male athletes than in female athletes compared with their sedentary controls. However, studies for the sex-specific cardiac remodeling related to strength exercises in Asian athletes are rare.

**Methods:**

This study included 580 men and 79 women, with an average age of 25 years, for a 6-month military training program in Taiwan. Both men and women attended a 2-min sit-up test to assess muscular strength after the training. The test performance falling one standard deviation above the mean (16%) was to define the superior eliteness of athletes. Cardiac structure and function were investigated by electrocardiography and echocardiography for men and women. Multiple logistic regression was used to determine the predictors of elite athlete status.

**Results:**

In men, greater QTc interval, left ventricular mass adjusted to body surface area (LVMI), lateral mitral E'/A' ratio and right ventricular systolic pressure, and lower diastolic blood pressure were independent predictors of elite strength athletes in the sit-up test [odds ratio (OR) and 95% confidence intervals: 1.01 (1.00, 1.02), 1.02 (1.00, 1.04), 1.45 (1.06, 1.98), 1.13 (1.06, 1.23), and 0.96 (0.93, 0.99), respectively. In contrast, in women, the greater right ventricular outflow tract dimension was the only independent predictor of elite strength athletes in the sit-up test [OR: 1.26 (1.04, 1.53)].

**Conclusions:**

In the 2-min sit-up test, cardiac characteristics differ between elite male and female athletes. While greater QTc interval, LVMI, and diastolic function of left ventricle predict the eliteness of male strength athletes, greater right ventricular chamber size characterizes elite female strength athletes.

## Introduction

Changes in cardiac structure and diastolic function in response to exercises are regarded as a benign process. Greater left ventricular mass and diastolic function measured by echocardiography are more commonly observed in elite athletes for either endurance or strength exercises than in sedentary controls (1, 2). However, some cardiac adaptations may vary based on the main exercise athletes perform. With regard to the left ventricular (LV) diastolic function, assessed by the E/A ratio utilizing mitral inflow doppler, it is slightly increased or normal in athletes compared to sedentary controls (3–5). While the LV diastolic function is evaluated by tissue doppler imaging of septal or lateral wall motion for the E'/A' ratio, it is greater in athletes due to an enhanced peak early E' or a reduced late atrial A' velocity than controls (4–6). To date, these findings on athletes' hearts were mainly obtained in studies from Western populations.

Previous reports have unveiled racial and sex differences in physiological cardiac adaptions to exercise and LV pressure overload (7–9). For a given level of physical training, athletes of African/Afro-Caribbean descent reveal more marked changes to cardiac structure than Caucasian athletes (8, 9), possibly due in part to genetic variations. In addition, greater changes in cardiac structure and function in response to regular physical training have been observed in male athletes than in female athletes compared to their sedentary controls (10). However, rare studies have investigated cardiovascular health between male and female Asian athletes. Moreover, most of the prior studies compared the physiological cardiac remodeling of elite athletes to sedentary controls but rarely to those under a similar training program. Therefore, this study aimed to compare the cardiac structure and function between elite male and female athletes for a 2-min sit-up exercise relative to their physical controls in the military in Taiwan.

## Methods

### Study Population

The Cardiorespiratory Fitness and Hospitalization Events in Armed Forces study (CHIEF Heart Study) included 580 men and 79 women, aged 18-34 years, from the ROC Army Huadong Defense Command Base in Taiwan and took place in January 2019 (11). All participants underwent the same training program for a 3-km run at 6:00 a.m. and 16:00 p.m., respectively, and were asked to complete the run in a limited time of 20 min. In addition, all participants had to perform at least 20 successive push-ups and subsequently 20 successive sit-ups following each run within 30 min. In July 2019, all of them attended a midterm exam for an evaluation of their muscular strength fitness, assessed by the 2-min sit-up exercise test. After the midterm physical exam, all received an annual health examination, including a self-report regarding their habits of tobacco smoking and alcohol beverage consumption (active vs. former and never) in the Hualien Armed Forces General Hospital of Eastern Taiwan. All participants carried out 12-lead electrocardiography (ECG) and transthoracic echocardiography for assessing their cardiac structure and function. The study design of the CHIEF study has been described in detail previously (12–15).

### Anthropometric and Hemodynamic Measurements

The body height and body weight of each participant were measured in a standing position. Body surface area was calculated as 0.20247 × body height (m)^0.725^ × body weight (kg)^0.425^ based on the Dubois formula (16). Body mass index was calculated as body weight (kg) divided by square of body height (m^2^). The Resting Blood pressure and pulse rate of each participant were measured once over the right upper arm in a sitting position by an automated blood pressure monitor (FT-201, Parama-Tech Co Ltd, Fukuoka, Japan).

### Muscular Strength Measurements

The muscular strength of each participant was investigated by the performance of the 2-min sit-up exercise (17). Participants were prepared in a supine position and fixed their feet by the anchor on the pad floor with both hands attached to the ears. It was scored using a computerized infrared system merely when their upper trunk bent forward and elbows simultaneously touched the artificial sensors on both thighs. If either one of the hands left the ears temporarily, the score would not be counted by the supervisors.

### Electrocardiographic and Echocardiographic Measurements

The 12-lead ECG features are interpreted *via* the software of the Schiller AG CARDIOVIT MS-2015 (Baar, Switzerland). The quality of ECG was required to be visually interpretable and with a smooth baseline; otherwise, the ECG would be repeated for acceptable quality. The echocardiography used a 1-5 MHz transducer (iE33; Philips Medical Systems, Andover, MA, USA) and was performed by an experienced technician and verified by a certificated cardiologist at the Hualien-Armed Forces General Hospital (18).

Cardiac structure such as LV wall thickness and size were measured according to the recommendations of the American Society of Echocardiography (19, 20). The corrected formula of LV mass proposed by Devereux et al. (21) was defined at end diastole as “0.8 × {1.04 × [(LV internal diameter (LVIDd) + posterior wall thickness + interventricular septal thickness]^3^ – LVIDd^3^} + 0.6.” LV hypertrophy for men was defined as the LV mass index as adjusted by body surface area (LVMI) ≥ 116 g/m^2^ based on the Dubois formula (11). Right ventricular (RV) hypertrophy for men was defined as the anterior RV wall thickness in parasternal long-axis window > 5.2 mm at end diastole (13). Mitral inflow Doppler for the velocity of the diastolic wave (E wave and A wave) and the E/A ratio. Tissue Doppler imaging for the lateral mitral annulus movement velocity (E' and A') and the E'/A' ratio were used to assess LV diastolic function. RV systolic pressure was evaluated by the continuous wave Doppler for trans-tricuspid velocity in the four-chamber window.

### Statistical Analysis

Elite male and female athletes were defined as the 2-min sit-up test score falling one standard deviation above the mean (16%). The controls were their physically active counterparts, who did not achieve the level for elite athletes. Demographic, anthropometric, hemodynamic, ECG, and echocardiographic profiles of elite male and female athletes and sex-specific non-elite controls were presented as mean ± standard deviation for continuous variables and numbers (percentages) for categorical variables.

Continuous variables were compared by two-sample *t*-test and categorical variables were compared by chi-square or Fisher's exact test for men and women, respectively. Multiple logistic regressions were utilized to determine the odds ratio (OR) of the ECG and echocardiographic predictors of the elite athletes to non-elite controls in men and women. In addition, multiple linear regressions were also utilized to determine the independent predictors for sit-up numbers within 2 min in men and women. A two-tailed value of *P* < 0.05 was considered significant.

All analyses were performed using SAS version 9.4 (SAS Institute, Cary, NC, USA). This study was reviewed and approved by the Institutional Review Board of the Mennonite Christian Hospital (No. 16-05-008) in Taiwan and written informed consent was obtained from all participants.

## Results

### Clinical Features and Laboratory Findings

In total, 93 (16.0%) of the 580 military men and 14 (17.7%) of the 79 military women who were classified as elite athletes performed >51 and 50 sit-ups within 2 min, as outlined in Table 1. In a study led by Ojeda et al. (22), the levels of very good or excellent young male and female athletes for sit-ups were consistent with the present study results. As compared with non-elite controls, elite strength athletes had similar levels of age, anthropometric parameters, and blood biochemistries except for lower diastolic blood pressure and greater serum levels of high-density lipoprotein and creatinine for elite male athletes, and greater serum triglycerides for elite female athletes.

**Table 1:** Clinical characteristics of elite male and female strength athletes and non-elite controls in the 2-min sit-up exercise.

	**Male athletes (*****N*** **=** **580)**				**Female athletes (*****N*** **=** **79)**		
	**Elite**	**Non-elite controls**	***P*-value**		**Elite**	**Non-elite controls**	***P*-value**
	**(≥51 numbers)**	**(<51 numbers)**			**(≥50 numbers)**	**(<50 numbers)**	
	**(*N* = 93)**	**(N = 487)**			**(*N* = 14)**	**(*N* = 65)**	
Age (years)	25.13 ± 3.69	25.21 ± 3.73	0.85		23.79 ± 2.94	24.05 ± 2.90	0.76
2-min sit-ups (numbers)	59.34 ± 6.85	43.26 ± 5.59	<0.001		53.57 ± 4.92	36.29 ± 6.06	<0.001
(Range: min-max, median)	(51-80, 60)	(20-50, 44)			(50-62, 50)	(20-45, 36)	
Height (cm)	171.57 ± 5.53	172.16 ± 5.62	0.35		160.52 ± 7.78	160.17 ± 4.82	0.82
Weight (kg)	71.09 ± 10.02	72.5 ± 11.59	0.24		60.67 ± 11.09	59.39 ± 7.87	0.61
Body surface area (m^2^)	1.83 ± 0.14	1.85 ± 0.16	0.24		1.64 ± 0.18	1.62 ± 0.11	0.65
Waist circumference (cm)	80.59 ± 8.01	82.21 ± 8.93	0.24		74.92 ± 10.27	75.65 ± 7.19	0.75
Systolic blood pressure (mmHg)	116.14 ± 11.35	117.29 ± 11.91	0.38		110.57 ± 13.11	104.88 ± 10.11	0.07
Diastolic blood pressure (mmHg)	65.98 ± 8.70	68.35 ± 8.69	0.01		64.64 ± 10.73	63.11 ± 6.32	0.47
**Blood test**
Serum creatinine (mg/dl)	0.97 ± 0.11	0.94 ± 0.10	0.04		0.68 ± 0.08	0.69 ± 0.07	0.92
Total cholesterol (mg/dl)	170.67 ± 34.37	168.07 ± 33.10	0.49		173.29 ± 23.99	167.28 ± 25.30	0.41
HDL-C (mmol/L)	51.41 ± 11.21	48.98 ± 9.22	0.02		57.43 ± 8.74	63.23 ± 11.70	0.08
LDL-C (mmol/L)	104.56 ± 30.45	104.08 ± 30.32	0.88		95.79 ± 21.23	91.57 ± 20.62	0.49
Serum triglyceride (mg/dl)	94.40 ± 68.46	97.50 ± 67.44	0.68		91.00 ± 60.68	59.58 ± 20.91	<0.001
Fasting plasma glucose (mg/dl)	93.66 ± 9.64	93.05 ± 8.87	0.55		95.07 ± 9.77	91.35 ± 7.42	0.11
Hemoglobin (g/dl)	15.22 ± 0.96	15.29 ± 0.90	0.55		12.65 ± 1.38	12.97 ± 0.96	0.31
Current tobacco smoking	46 [49.5]	208 [42.7]	0.22		2 [14.3]	10 [15.4]	0.91

*Continuous variables are expressed as mean ± standard deviation, and categorical variables as n [%]*.

*HDL-C, high-density lipoprotein cholesterol; LDL-C, low-density lipoprotein cholesterol*.

### Electrocardiographic Features

As indicated by Table 2, in men, elite strength athletes had a significantly greater corrected QT (QTc) interval defined by the Bazett's formula (393.10 ± 27.15 vs. 387.43 ± 21.36 ms, *p* = 0.02) and a higher prevalence of QTc interval prolongation > 480 ms (3.2 vs. 0.2%, *p* = 0.001) (23), compared to non-elite controls. In women, there were no significant differences in any ECG characteristics between elite strength athletes and non-elite controls.

**Table 2:** Electrocardiographic characteristics of elite male and female strength athletes and non-elite controls in the 2-min sit-up exercise.

	**Male athletes (*****N*** **=** **580)**				**Female athletes (*****N*** **=** **79)**		
	**Elite**	**Non-elite controls**	***P*-value**		**Elite**	**Non-elite controls**	***P*-value**
	**(≥51 numbers)**	**(<51 numbers)**			**(≥50 numbers)**	**(<50 numbers)**	
	**(*N* = 93)**	**(N = 487)**			**(*N* = 14)**	**(*N* = 65)**	
Heart rate (beats/min)	65.82 ± 11.67	65.97 ± 10.62	0.89		67.50 ± 11.07	68.74 ± 9.46	0.66
P duration (ms)	107.88 ± 13.15	105.56 ± 14.39	0.14		99.74 ± 11.50	99.12 ± 13.72	0.87
PR interval (ms)	157.38 ± 26.14	154.45 ± 17.94	0.18		147.75 ± 21.17	151.86 ± 20.10	0.49
QRS duration (ms)	98.19 ± 10.18	97.30 ± 10.00	0.43		84.29 ± 5.42	85.88 ± 7.80	0.47
QTc interval (ms)	393.10 ± 27.15	387.43 ± 21.36	0.02		408.14 ± 17.33	411.89 ± 30.62	0.66
QRS axis (degree)	64.76 ± 23.86	65.29 ± 28.42	0.86		72.43 ± 13.60	64.11 ± 34.79	0.38
ECG based LVH (%)	59 [63.4]	287[58.9]	0.41		1 [7.1]	2 [3.1]	0.47
ECG based RVH (%)	15 [16.1]	80 [16.4]	0.94		0 [0.0]	0 [0.0]	–
Sinus bradycardia (%)	30 [32.3]	136 [27.9]	0.39		2 [14.3]	12 [18.5]	0.71
Ectopic P rhythm (%)	1 [1.1]	21 [4.3]	0.13		0 [0.0]	3 [4.6]	0.41
Left atrial enlargement (%)	11 [11.8]	83 [17.0]	0.21		1 [7.1]	12 [18.5]	0.30
First degree atrioventricular block (%)	3 [3.2]	8 [1.6]	0.30		0 [0.0]	2 [3.1]	0.50
Left axis deviation (%)	2 [2.2]	3 [0.6]	0.14		0 [0.0]	3 [4.6]	0.41
Right axis deviation (%)	5 [5.4]	50 [10.3]	0.14		2 [14.3]	9 [13.8]	0.96
Complete right bundle branch block (%)	2 [2.2]	14 [2.9]	0.69		0 [0.0]	0 [0.0]	–
Incomplete right bundle branch block (%)	10 [10.8]	28 [5.7]	0.07		0 [0.0]	0 [0.0]	–
QT interval prolongation (%)	2 [2.2]	7 [1.4]	0.61		0 [0.0]	1 [1.5]	0.64
QTc prolongation (%)	3 [3.2]	1 [0.2]	0.001		0 [0.0]	2 [3.1]	0.50
T wave inversion (%)	2 [2.2]	15 [3.1]	0.62		0 [0.0]	4 [6.2]	0.34

*Categorical variables are expressed as N [%]*.

### Echocardiographic Findings

In Table 3, in men, elite strength athletes had greater LVMI (80.9 ± 11.7 vs. 77.5 ± 12.0 g/m^2^, p = 0.001), RV systolic pressure (29.5 ± 4.15 vs. 27.8 ± 12.0 mmHg, p = 0.001), and lateral mitral E'/A' (2.32 ± 0.70 vs. 2.11 ± 0.71, p = 0.01) compared to non-elite controls. In contrast, in women, elite strength athletes were found to have greater aortic root dimension (30.2 ± 3.70 vs. 26.9 ± 2.52 mm, p <0.01), and RV outflow tract dimension compared to non-elite controls (28.5 ± 4.04 vs. 25.7 ± 3.46 mm, p = 0.01).

**Table 3:** Echocardiographic characteristics of elite male and female strength athletes and non-elite controls in the 2-min sit-up exercise.

	**Male athletes (N** **=** **580)**				**Female athletes (N** **=** **79)**		
	**Elite**	**Non-elite controls**	**P-value**		**Elite**	**Non-elite controls**	**P-value**
	**(≥51 numbers)**	**(<51 numbers)**			**(≥50 numbers)**	**(<50 numbers)**	
	**(N = 93)**	**(N = 487)**			**(N = 14)**	**(N = 65)**	
Aortic valve open (mm), PLAX	20.53 ± 1.72	20.35 ± 1.85	0.38		19.93 ± 2.05	18.66 ± 1.81	0.02
Aortic root dimension (mm), PALX	30.09 ± 2.45	29.83 ± 2.74	0.39		30.21 ± 3.70	26.92 ± 2.52	<0.001
LV posterior wall (mm), PLAX	8.41 ± 0.82	8.29 ± 0.83	0.20		7.07 ± 0.82	7.11 ± 0.75	0.83
LV internal dimension in diastole (mm), PLAX	49.60 ± 3.37	49.07 ± 3.48	0.17		44.64 ± 3.02	44.52 ± 2.88	0.88
LV internal dimension in systole (mm), PLAX	31.38 ± 3.08	30.37 ± 3.22	0.006		30.86 ± 3.67	28.12 ± 2.84	0.003
Interventricular septum, (mm), PLAX	8.55 ± 0.80	8.48 ± 0.89	0.52		7.29 ± 0.99	7.28 ± 0.78	0.97
RV wall thickness (mm), PLAX	4.65 ± 0.63	4.69 ± 0.59	0.54		4.49 ± 0.73	4.33 ± 0.45	0.30
RV outflow tract dimension in diastole (mm), PLAX	26.47 ± 3.72	26.30 ± 3.99	0.70		28.50 ± 4.04	25.72 ± 3.46	0.01
Left atrial dimension (mm), PLAX	32.97 ± 3.34	32.83 ± 4.00	0.74		33.21 ± 3.74	30.98 ± 4.94	0.11
LV mass (gm)	148.64 ± 24.01	144.35 ± 27.16	0.15		100.87 ± 21.93	100.40 ± 18.84	0.93
LV mass index (gm/m^2^)	80.93 ± 11.73	77.52 ± 12.00	0.01		61.11 ± 9.19	61.65 ± 9.28	0.84
LV hypertrophy	1 [1.1]	5 [1.0]	0.96		0 [0.0]	2 [3.1]	0.50
RV hypertrophy	2 [2.2]	26 [5.3]	0.18		2 [14.3]	2 [3.1]	0.08
LV ejection fraction (%), PLAX	62.52 ± 5.36	62.50 ± 5.32	0.97		63.43 ± 5.68	61.20 ± 4.91	0.13
Tricuspid valve prolapse, PSAX	19 [51.4]	89 [49.7]	0.85		1 [20.0]	12 [63.2]	0.08
Aortic regurgitation ≥ mild grade	1 [1.1]	3 [0.6]	0.62		2 [14.3]	2 [3.1]	0.08
Mitral regurgitation ≥ mild grade	82 [88.2]	419 [86.0]	0.58		13 [92.9]	56 [86.2]	0.49
Pulmonary regurgitation ≥ mild grade	71 [76.3]	346 [71.0]	0.29		11 [78.6]	49 [75.4]	0.80
Tricuspid regurgitation ≥ mild grade	87 [93.5]	451 [92.6]	0.74		14 [100.0]	60 [92.3]	0.28
RV systolic pressure (mmHg)	29.53 ± 4.15	27.80 ± 3.73	<0.001		28.21 ± 3.19	27.49 ± 3.75	0.50
Mitral inflow power Doppler E-wave (m/s)	89.64 ± 14.62	87.32 ± 14.45	0.15		94.35 ± 10.49	96.75 ± 13.75	0.54
Mitral inflow power Doppler A-wave (m/s)	49.25 ± 9.10	49.25 ± 9.95	0.99		47.67 ± 8.32	50.85 ± 10.43	0.29
E/A ratio	1.88 ± 0.49	1.84 ± 0.47	0.41		2.03 ± 0.44	1.98 ± 0.50	0.68
Mitral lateral annulus tissue Doppler E' (cm/s)	18.89 ± 4.21	18.20 ± 4.04	0.13		19.75 ± 3.40	18.88 ± 3.90	0.44
Mitral lateral annulus tissue Doppler A' (cm/s)	8.50 ± 1.90	9.33 ± 4.99	0.11		9.52 ± 2.75	8.92 ± 2.19	0.37
E'/A' ratio	2.32 ± 0.70	2.11 ± 0.71	0.01		2.23 ± 0.80	2.23 ± 0.68	0.97

*Continuous variables are expressed as mean ± SD (standard deviation), and categorical variables as N [%]*.

*LV, left ventricle; RV, right ventricle;PLAX, echocardiographic parasternal long axis view; PSAX, echocardiographic parasternal short axis view*.

### Echocardiographic Predictors of Elite Strength Athletes

In Table 4, the multiple logistic regression results showed that, in men, greater QTc interval, LVMI, RV systolic pressure and lateral mitral E'/A', and lower diastolic blood pressure were independent predictors of elite strength athletes [odds ratio (OR): 1.01 [95% confidence intervals (CI): 1.00, 1.02], 1.02 (95% CI: 1.00, 1.04), 1.13 (95% CI: 1.06, 1.20), 1.45 (95%CI: 1.06, 1.98) and 0.96 (95% CI: 0.93, 0.99), respectively]. In contrast, RV outflow tract dimension was the only independent predictor of elite strength athletes in women [OR: 1.26 (95% CI: 1.04, 1.53)].

**Table 4:** Multivariable logistic regression analysis for elite male and female strength athletes.

	**Elite male athletes (*****N*** **=** **580)**				**Elite female athletes (*****N*** **=** **79)**		
**Characteristics**	**OR**	**95% CI**	***P*-value**		**OR**	**95% CI**	***P*-value**
Systolic blood pressure	1.007	0.984-1.031	0.55		1.058	0.984-1.137	0.12
Diastolic blood pressure	0.962	0.931-0.994	0.02		0.988	0.894-1.091	0.80
QTc interval	1.012	1.002-1.022	0.01		1.003	0.977-1.031	0.80
LV mass index	1.020	1.000-1.040	0.04		0.980	0.911-1.055	0.59
RV outflow tract dimension	1.016	0.958-1.078	0.59		1.262	1.042-1.529	0.01
RV systolic pressure	1.126	1.060-1.196	<0.001		1.016	0.836-1.233	0.87
E'/A' ratio	1.450	1.063-1.978	0.01		1.176	0.475-2.911	0.72

*Data are presented as odds ratios (OR) and 95% CI (confidence intervals) using multiple logistic regression*.

*LV, left ventricle; RV, right ventricle*.

As shown in Table 5, the results of multiple linear regressions were consistent for LVMI, RV systolic pressure, and lateral mitral E'/A' in men [β: 0.068, 0.28, and 1.61, respectively; all *p*-values ≤ 0.01] and RV outflow tract dimension in women [β: 0.28; *p* = 0.01], except that the correlations of diastolic blood pressure and QTc interval with sit-up numbers were not significant.

**Table 5:** Multivariable linear regression analysis for sit-up numbers in men and women.

	**Sit-up numbers in men (*****N*** **=** **580)**				**Sit-up numbers in women (*****N*** **=** **79)**		
**Characteristics**	**β**	**95% CI**	***P*-value**		**β**	**95% CI**	***P*-value**
Systolic blood pressure	−0.012	−0.079-0.055	0.72		0.144	−0.086-0.375	0.21
Diastolic blood pressure	−0.060	−0.150-0.030	0.19		−0.130	−0.470-0.211	0.44
QTc interval	0.013	−0.016-0.043	0.37		−0.033	−0.108-0.041	0.37
LV mass index	0.068	0.011-0.124	0.01		−0.023	−0.243-0.196	0.83
RV outflow tract dimension	0.031	−0.139-0.201	0.72		0.676	0.118-1.233	0.01
RV systolic pressure	0.284	0.112-0.456	0.001		0.127	−0.418-0.672	0.64
E'/A' ratio	1.608	0.665-2.552	0.001		−0.191	−3.065-2.684	0.89

*Data are presented as odds ratios (OR) and 95% CI (confidence intervals) using multiple logistic regression*.

*LV, left ventricle; RV, right ventricle*.

## Discussion

This study is the largest to date for the Asian population to show the sex-specific cardiac structure and function of elite athletes compared to physically active controls and to determine the ECG and echocardiographic predictors of eliteness in the 2-min sit-ups. The main findings in the present study were that in men, greater QTc interval, LVMI, LV diastolic function, and RV systolic pressure, and lower diastolic blood pressure were found as independent predictors of falling in the elite strength category. By contrast, in women, the greater RV outflow tract dimension was the only independent predictor of being in the elite strength category.

Strength exercise training has been found to increase left ventricular mass, but not alter LV chamber size in both male and female athletes in prior studies (6, 9). Exercise related LV hypertrophy could be induced by an elongation of cardiac muscle cells resistant to chronic pressure overload, which is regulated partially by the renin-angiotensin system (24, 25). Prior studies have found greater changes in cardiac structure and function following strength exercises in male athletes than in female athletes compared to their sedentary controls (9, 10). The present study further confirmed the concept that greater LVMI and lateral mitral E'/A' ratio independently predict the performance of the sit-up exercise in physically active men but not women. The mechanisms for more marked cardiac structure changes in male athletes could be reasoned in part by an increased QTc interval, indicating a longer cardiac systolic phase and possibly leading to greater LVMI. By contrast, for physically active women, both elite and non-elite athletes had lower blood pressure at rest, causing lower LV pressure overload and thus less physiological cardiac remodeling.

For the cardiovascular structural changes in women, the present study revealed that elite female strength athletes had greater aortic root and RV outflow tract dimensions, implying greater stroke volume compared with non-elite controls. In addition, elite female strength athletes tended to have a higher systolic blood pressure than non-elite controls, whereas elite male strength athletes had lower diastolic blood pressure. The findings clarified that the hemodynamic changes related to the sit-up test performance might differ by sex and thus influence the cardiac structure remodeling. Moreover, prior studies showed that in athletes, RV chamber size was positively associated with RV systolic pressure (26), which was not observed in both our male and female strength athletes. Whether these conflicts were due to racial/ethnic differences or the presence of unrecognized confounders in prior studies needs further investigation.

### Strengths and Limitations

A strength of the present study is that participants were enrolled from the military under the same training program. In addition, the army base was a closed system, which implies that consistency in living circumstances. Third, the performance of the sit-up exercise was standardized, which could reduce observer bias.

Limitations arise from the fact that strength capacity was assessed only by the 2-min sit-up test, meaning the cardiac structure and function results might not be applied appropriately to the athletes of other exercise modalities. Second, there were only 79 military women in the present study, meaning results might lack sufficient power to detect some important predictors of elite athletes for sit-ups. Finally, we did not have the 12-lead ECG and echocardiographic data prior to training, and the ECG and echocardiographic changes could not be compared between elite athletes and non-elite controls.

## Conclusion

Our study revealed that in the 2-min sit-up test, the cardiac characteristics differ between elite male and female athletes compared with their physically active non-elite controls. While greater QTc interval, RV systolic pressure, LV diastolic function, and LVMI predict elite strength athletes in men, greater RV chamber size characterizes elite strength athletes in women.

## Data Availability Statement

The raw data supporting the conclusions of this article will be made available by the authors, without undue reservation.

## Ethics Statement

This study was reviewed and approved by the Institutional Review Board of the Mennonite Christian Hospital (No. 16-05-008) in Taiwan. The patients/participants provided their written informed consent to participate in this study.

## Author Contributions

Y-KL wrote the paper. K-ZT made the statistical analyses. C-LH and J-TL raised critical comments for the paper. G-ML was the principal investigator for the study. All authors contributed to the article and approved the submitted version.

## Funding

This study was supported by the Medical Affairs Bureau Ministry of National Defense and Hualien Armed Forces General Hospital, Taiwan, under the grants MND-MAB-110-148, MND-MAB-D-11115, HAFGH-D-110008, and HAFGH-D-11115.

## Conflict of Interest

The authors declare that the research was conducted in the absence of any commercial or financial relationships that could be construed as a potential conflict of interest.

## Publisher's Note

All claims expressed in this article are solely those of the authors and do not necessarily represent those of their affiliated organizations, or those of the publisher, the editors and the reviewers. Any product that may be evaluated in this article, or claim that may be made by its manufacturer, is not guaranteed or endorsed by the publisher.
