# Supplementary material for: Athlete's Heart Assessed by Sit-Up Strength Exercises in Military Men and Women: The CHIEF Heart Study
Source: Front Cardiovasc Med. 2022 Jan 26;8:737607. doi: 10.3389/fcvm.2021.737607 (PMC8826563; doi:10.3389/fcvm.2021.737607)
Supplement: Supplementary file 1 [file Data_Sheet_1.docx]

**Supplemental Table 1.** Clinical Characteristics of Elite Male and Female Strength Athletes and Non-Elite Controls in the 2-Minute Sit-Up Exercise

|  | Male Athletes  (N =580) | | |  | Female Athletes  (N =79) | | |
| --- | --- | --- | --- | --- | --- | --- | --- |
| Clinical characteristics | Elite  (≥51 numbers)  (N =93) | Non-elite controls  (<51 numbers)  (N =487) | p-value |  | Elite  (≥50 numbers)  (N =14) | Non-elite controls  (<50 numbers)  (N =65) | p-value |
| Age (years) | 25.13 ± 3.69 | 25.21 ± 3.73 | 0.85 |  | 23.79 ± 2.94 | 24.05 ± 2.90 | 0.76 |
| (Range: min – max, median) | (19 – 33, 25) | (19 – 34, 25) |  |  | (21 – 30, 23) | (20 – 30, 24) |  |
| 2-min sit-ups (numbers) | 59.34 ± 6.85 | 43.26 ± 5.59 | <0.001 |  | 53.57 ± 4.92 | 36.29 ± 6.06 | <0.001 |
| (Range: min – max, median) | (51 – 80, 60) | (20 – 50, 44) |  |  | (50 – 62, 50) | (20 – 45, 36) |  |
| Height (cm) | 171.57 ± 5.53 | 172.16 ± 5.62 | 0.35 |  | 160.52 ± 7.78 | 160.17 ± 4.82 | 0.82 |
| (Range: min – max, median) | (158.7 – 186.1, 171.1) | (156.3 – 187.6, 172.3) |  |  | (151.0 – 176.0, 158.8) | (149.7 – 174.1, 159.5) |  |
| Weight (kg) | 71.09 ± 10.02 | 72.59 ± 11.59 | 0.24 |  | 60.67 ± 11.09 | 59.39 ± 7.87 | 0.61 |
| (Range: min – max, median) | (48.9 – 96.0, 70.1) | (48.8 – 118, 71.9) |  |  | (42.6 – 77.6, 62.5) | (44.4 – 80.0, 58.2) |  |
| Body surface area (m^2^) | 1.83 ± 0.14 | 1.85 ± 0.16 | 0.24 |  | 1.64 ± 0.18 | 1.62 ± 0.11 | 0.65 |
| (Range: min – max, median) | (1.48 – 2.18, 1.83) | (1.47 – 2.44, 1.85) |  |  | (1.37 – 1.94, 1.68) | (1.38 – 1.93, 1.61) |  |
| Waist circumference (cm) | 80.59 ± 8.01 | 82.21 ± 8.93 | 0.24 |  | 74.92 ± 10.27 | 75.65 ± 7.19 | 0.75 |
| (Range: min – max, median) | (65 – 100, 80) | (60 – 109, 81) |  |  | (58 – 93, 75) | (64 – 92, 74) |  |
| Systolic blood pressure (mmHg) | 116.14 ± 11.35 | 117.29 ± 11.91 | 0.38 |  | 110.57 ± 13.11 | 104.88 ± 10.11 | 0.07 |
| (Range: min – max, median) | (85 – 150, 116) | (84 – 166, 117) |  |  | (99 – 147, 106) | (86 – 133, 102) |  |
| Diastolic blood pressure (mmHg) | 65.98 ± 8.70 | 68.35 ± 8.69 | 0.01 |  | 64.64 ± 10.73 | 63.11 ± 6.32 | 0.47 |
| (Range: min – max, median) | (40 – 88, 66) | (42 – 106, 68) |  |  | (42 – 86, 63) | (50 – 87, 62) |  |
| Blood test |  |  |  |  |  |  |  |
| Serum creatinine (mg/dL) | 0.97 ± 0.11 | 0.94 ± 0.10 | 0.04 |  | 0.68 ± 0.08 | 0.69 ± 0.07 | 0.92 |
| (Range: min – max, median) | (0.73 – 1.43, 0.97) | (0.64 – 1.47, 0.94) |  |  | (0.57 – 0.83, 0.70) | (0.48 – 0.88, 0.68) |  |
| Total cholesterol (mg/dL) | 170.67 ± 34.37 | 168.07 ± 33.10 | 0.49 |  | 173.29 ± 23.99 | 167.28 ± 25.30 | 0.41 |
| (Range: min – max, median) | (94 – 303, 167) | (85 – 346, 165) |  |  | (128 – 206, 172) | (116 – 232, 164) |  |
| HDL-C (mmol/L) | 51.41 ± 11.21 | 48.98 ± 9.22 | 0.02 |  | 57.43 ± 8.74 | 63.23 ± 11.70 | 0.08 |
| (Range: min – max, median) | (27 – 79, 50) | (26 – 82, 49) |  |  | (43 – 73, 58) | (30 – 98, 63) |  |
| LDL-C (mmol/L) | 104.56 ± 30.45 | 104.08 ± 30.32 | 0.88 |  | 95.79 ± 21.23 | 91.57 ± 20.62 | 0.49 |
| (Range: min – max, median) | (35 – 199, 101) | (30 – 254, 100) |  |  | (60 – 145, 96) | (45 – 165, 90) |  |
| Serum triglyceride (mg/dL) | 94.40 ± 68.46 | 97.50 ± 67.44 | 0.68 |  | 91.00 ± 60.68 | 59.58 ± 20.91 | <0.001 |
| (Range: min – max, median) | (34 – 547, 77) | (31 – 566, 77) |  |  | (32 – 213, 62) | (25 – 157, 59) |  |
| Fasting plasma glucose (mg/dL) | 93.66 ± 9.64 | 93.05 ± 8.87 | 0.55 |  | 95.07 ± 9.77 | 91.35 ± 7.42 | 0.11 |
| (Range: min – max, median) | (66 – 143, 94) | (65 – 155, 93) |  |  | (88 – 126, 91) | (64 – 105, 91) |  |
| Hemoglobin (g/dL) | 15.22 ± 0.96 | 15.29 ± 0.90 | 0.55 |  | 12.65 ± 1.38 | 12.97 ± 0.96 | 0.31 |
| (Range: min – max, median) | (12.6 – 17.6, 15.2) | (12.1 – 17.6, 15.3) |  |  | (9.7 – 14.5, 12.9) | (10.1 – 14.7, 13.0) |  |

Continuous variables are expressed as mean ± standard deviation, and categorical variables as n [%]

Abbreviations: HDL-C, high-density lipoprotein cholesterol; LDL-C, low-density lipoprotein cholesterol.

**Supplemental Table 2.** Electrocardiographic Characteristics of Elite Male and Female Strength Athletes and Non-Elite Controls in the 2-Minute Sit-Up Exercise

|  | Male Athletes  (N =580) | | |  | Female Athletes  (N =79) | | |
| --- | --- | --- | --- | --- | --- | --- | --- |
| ECG characteristics | Elite  (≥51 numbers)  (N =93) | Control  (<51 numbers)  (N =487) | p-value |  | Elite  (≥50 numbers)  (N =14) | Control  (<50 numbers)  (N =65) | p-value |
| Heart rate (beats/min) | 65.82 ± 11.67 | 65.97 ± 10.62 | 0.89 |  | 67.50 ± 11.07 | 68.74 ± 9.46 | 0.66 |
| (Range: min – max, median) | (42 – 105, 65) | (38 – 105, 65) |  |  | (52 – 95, 67) | (50 – 90, 70) |  |
| P duration (ms) | 107.88 ± 13.15 | 105.56 ± 14.39 | 0.14 |  | 99.74 ± 11.50 | 99.12 ± 13.72 | 0.87 |
| (Range: min – max, median) | (68 – 148, 106) | (56 – 184, 106) |  |  | (78 – 118, 106) | (54 – 122, 104) |  |
| PR interval (ms) | 157.38 ± 26.14 | 154.45 ± 17.94 | 0.18 |  | 147.75 ± 21.17 | 151.86 ± 20.10 | 0.49 |
| (Range: min – max, median) | (96 – 342, 157) | (74 – 248, 157) |  |  | (98 – 148, 157) | (110 – 208, 156) |  |
| QRS duration (ms) | 98.19 ± 10.18 | 97.30 ± 10.00 | 0.43 |  | 84.29 ± 5.42 | 85.88 ± 7.80 | 0.47 |
| (Range: min – max, median) | (84 – 140, 96) | (70 – 140, 96) |  |  | (78 – 94, 84) | (64 – 102, 86) |  |
| QTc interval (ms) | 393.10 ± 27.15 | 387.43 ± 21.36 | 0.02 |  | 408.14 ± 17.33 | 411.89 ± 30.62 | 0.66 |
| (Range: min – max, median) | (346 – 503, 391) | (338 – 494, 386) |  |  | (384 – 438, 405) | (354 – 526, 411) |  |
| QRS axis (degree) | 64.76 ± 23.86 | 65.29 ± 28.42 | 0.86 |  | 72.43 ± 13.60 | 64.11 ± 34.79 | 0.38 |
| (Range: min – max, median) | (-38 – 109, 68) | (-76 – 244, 70) |  |  | (58 – 107, 69) | (-66 – 119, 71) |  |

Categorical variables are expressed as N [%].

**Supplemental Table 3.** Echocardiographic Characteristics of Elite Male and Female Strength Athletes and Non-Elite Controls in the 2-Minute Sit-Up Exercise

|  | Male Athletes  (N =580) | | |  | Female Athletes  (N =79) | | |
| --- | --- | --- | --- | --- | --- | --- | --- |
| Echocardiographic characteristics | Elite  (≥51 numbers)  (N =93) | Control  (<51 numbers)  (N =487) | p-value |  | Elite  (≥50 numbers)  (N =14) | Control  (<50 numbers)  (N =65) | p-value |
| Aortic valve open (mm), PLAX | 20.53 ± 1.72 | 20.35 ± 1.85 | 0.38 |  | 19.93 ± 2.05 | 18.66 ± 1.81 | 0.02 |
| (Range: min – max, median) | (17 – 25, 20) | (15 – 25, 20) |  |  | (15 – 23, 20) | (15 – 24, 18) |  |
| Aortic root dimension (mm), PALX | 30.09 ± 2.45 | 29.83 ± 2.74 | 0.39 |  | 30.21 ± 3.70 | 26.92 ± 2.52 | <0.001 |
| (Range: min – max, median) | (23 – 36, 30) | (18 – 38, 30) |  |  | (25 – 38, 30) | (23 – 34, 27) |  |
| LV posterior wall (mm), PLAX | 8.41 ± 0.82 | 8.29 ± 0.83 | 0.20 |  | 7.07 ± 0.82 | 7.11 ± 0.75 | 0.83 |
| (Range: min – max, median) | (7 – 11, 8) | (6 – 11, 8) |  |  | (5 – 8, 7) | (6 – 9, 7) |  |
| LV internal dimension in diastole (mm), PLAX | 49.60 ± 3.37 | 49.07 ± 3.48 | 0.17 |  | 44.64 ± 3.02 | 44.52 ± 2.88 | 0.88 |
| (Range: min – max, median) | (41 – 57, 50) | (40 – 60, 49) |  |  | (41 – 51, 44) | (38 – 51, 45) |  |
| LV internal dimension in systole (mm), PLAX | 31.38 ± 3.08 | 30.37 ± 3.22 | 0.006 |  | 30.86 ± 3.67 | 28.12 ± 2.84 | 0.003 |
| (Range: min – max, median) | (25 – 39, 31) | (19 – 41, 30) |  |  | (23 – 36, 32) | (20 – 34, 28) |  |
| Interventricular septum, (mm), PLAX | 8.55 ± 0.80 | 8.48 ± 0.89 | 0.52 |  | 7.29 ± 0.99 | 7.28 ± 0.78 | 0.97 |
| (Range: min – max, median) | (7 – 11, 8) | (6 – 11, 8) |  |  | (5 – 9, 7) | (6 – 9, 7) |  |
| RV wall thickness (mm), PLAX | 4.65 ± 0.63 | 4.69 ± 0.59 | 0.54 |  | 4.49 ± 0.73 | 4.33 ± 0.45 | 0.30 |
| (Range: min – max, median) | (0.54 – 5.89, 4.71) | (0.54 – 5.95, 4.76) |  |  | (3.39 – 5.73, 4.61) | (3.19 – 5.45, 4.36) |  |
| RV outflow tract dimension in diastole (mm), PLAX | 26.47 ± 3.72 | 26.30 ± 3.99 | 0.70 |  | 28.50 ± 4.04 | 25.72 ± 3.46 | 0.01 |
| (Range: min – max, median) | (15 – 35, 26) | (16 – 36, 26) |  |  | (19 – 33, 29) | (19 – 33, 26) |  |
| Left atrial dimension (mm), PLAX | 32.97 ± 3.34 | 32.83 ± 4.00 | 0.74 |  | 33.21 ± 3.74 | 30.98 ± 4.94 | 0.11 |
| (Range: min – max, median) | (24 – 41, 33) | (20 – 52, 33) |  |  | (25 – 38, 33) | (22 – 58, 31) |  |
| LV mass (gm) | 148.64 ± 24.01 | 144.35 ± 27.16 | 0.15 |  | 100.87 ± 21.93 | 100.40 ± 18.84 | 0.93 |
| (Range: min – max, median) | (83.28 –197.78, 145.07) | (71.74 –230.52, 144.11) |  |  | (57.71 –140.01, 97.97) | (62.91 –150.23, 97.97) |  |
| LV mass index (gm/m^2^) | 80.93 ± 11.73 | 77.52 ± 12.00 | 0.01 |  | 61.11 ± 9.19 | 61.65 ± 9.28 | 0.84 |
| (Range: min – max, median) | (52.23 – 107.38, 80.12) | (45.57 – 128.92, 76.91) |  |  | (42.06 – 78.71, 59.60) | (40.86 – 88.44, 60.82) |  |
| LV ejection fraction (%), PLAX | 62.52 ± 5.36 | 62.50 ± 5.32 | 0.97 |  | 63.43 ± 5.68 | 61.20 ± 4.91 | 0.13 |
| (Range: min – max, median) | (50 – 70, 60) | (27 – 75, 60) |  |  | (55 – 70, 61) | (55 – 70, 60) |  |
| RV systolic pressure (mmHg) | 29.53 ± 4.15 | 27.80 ± 3.73 | <0.001 |  | 28.21 ± 3.19 | 27.49 ± 3.75 | 0.50 |
| (Range: min – max, median) | (20 – 40, 29) | (9 – 45, 28) |  |  | (25 – 37, 28) | (11 – 35, 28) |  |
| Mitral inflow power Doppler E-wave (m/sec) | 89.64 ± 14.62 | 87.32 ± 14.45 | 0.15 |  | 94.35 ± 10.49 | 96.75 ± 13.75 | 0.54 |
| (Range: min – max, median) | (49.4 – 122.0, 89.9) | (50.4 – 134.0, 86.7) |  |  | (75.0 – 144.0, 95.1) | (71.1 – 132.0, 97.2) |  |
| Mitral inflow power Doppler A-wave (m/sec) | 49.25 ± 9.10 | 49.25 ± 9.95 | 0.99 |  | 47.67 ± 8.32 | 50.85 ± 10.43 | 0.29 |
| (Range: min – max, median) | (28.8 – 79.3, 48.0) | (26.4 – 85.0, 48.0) |  |  | (35.8 – 60.0, 45.9) | (30.9 – 78.6, 50.0) |  |
| E/A ratio | 1.88 ± 0.49 | 1.84 ± 0.47 | 0.41 |  | 2.03 ± 0.44 | 1.98 ± 0.50 | 0.68 |
| (Range: min – max, median) | (1.06 – 3.44, 1.85) | (0.73 – 3.81, 1.76) |  |  | (1.33 – 2.93, 1.96) | (1.15 – 3.81, 1.94) |  |
| Mitral lateral annulus tissue Doppler E’ (cm/sec) | 18.89 ± 4.21 | 18.20 ± 4.04 | 0.13 |  | 19.75 ± 3.40 | 18.88 ± 3.90 | 0.44 |
| (Range: min – max, median) | (9.6 – 31.0, 18.9) | (8.4 – 34.2, 18.0) |  |  | (15.4 – 26.4, 18.7) | (12.4 – 31.7, 18.4) |  |
| Mitral lateral annulus tissue Doppler A’ (cm/sec) | 8.50 ± 1.90 | 9.33 ± 4.99 | 0.11 |  | 9.52 ± 2.75 | 8.92 ± 2.19 | 0.37 |
| (Range: min – max, median) | (5.3 – 14.3, 8.2) | (4.2 – 17.9, 8.7) |  |  | (6.2 – 14.7, 8.5) | (4.7 – 17.3, 8.5) |  |
| E’/A’ ratio | 2.32 ± 0.70 | 2.11 ± 0.71 | 0.01 |  | 2.23 ± 0.80 | 2.23 ± 0.68 | 0.97 |
| (Range: min – max, median) | (1.13 – 4.60, 2.27) | (0.26 – 5.26, 2.03) |  |  | (1.27 – 4.23, 2.02) | (1.09 – 4.37, 2.09) |  |

Continuous variables are expressed as mean ± SD (standard deviation), and categorical variables as N [%].

Abbreviations: left ventricle, LV; right ventricle, RV; echocardiographic parasternal long axis view, PLAX; echocardiographic parasternal short axis view, PSAX
